# Clinical outcomes of surgical treatment for end-stage ankle osteoarthritis in patients aged ≥ 75 years: a multicenter, retrospective study

**DOI:** 10.1186/s13018-023-03734-4

**Published:** 2023-03-25

**Authors:** Kentaro Amaha, Satoshi Yamaguchi, Atsushi Teramoto, Yohei Kawasaki, Yuki Shiko, Nobuto Kitamura

**Affiliations:** 1 Department of Orthopedic Surgery, St Luke’s International Hospital, Akashi-cho, Chuo-ku, Tokyo, Japan; 2 Graduate School of Global and Transdisciplinary Studies, Chiba University, Inohana, Chuo-ku, Chiba-shi, Chiba, Japan; 3 Department of Orthopaedic Surgery, Graduate School of Medical and Pharmaceutical Sciences, Chiba University, Inohana, Chuo-ku, Chiba-shi, Chiba, Japan; 4 Department of Orthopaedic Surgery, Sapporo Medical University School of Medicine, Chuo-ku, Sapporo, Hokkaido Japan; 5 Biostatistics Section, Clinical Research Center, Chiba University Hospital, Inohana, Chuo-ku, Chiba, Japan

**Keywords:** Ankle osteoarthritis, Ankle arthrodesis, Elderly patients, Total ankle arthroplasty

## Abstract

**Background:**

This study aimed to clarify the clinical outcomes of surgical treatment for end-stage ankle osteoarthritis in patients aged ≥ 75 years and compare these outcomes with those of patients aged < 75 years.

**Methods:**

A total of 148 patients, including 65 who underwent total ankle arthroplasty and 83 who underwent ankle arthrodesis, were retrospectively surveyed. Clinical outcomes were assessed preoperatively and at the last follow-up using the Japanese Society for Surgery of the Foot Hindfoot Scale and a self-administered foot evaluation questionnaire (SAFE-Q). Patient characteristics, including age, sex, body mass index, radiographic severity, and follow-up period, were also assessed. The patients were divided into older (≥ 75 years) and younger (< 75 years) age groups. Improvements in outcomes were then compared between age groups using univariate analysis and analysis of covariance adjusted for patient characteristics. Total ankle arthroplasty and ankle arthrodesis were analyzed separately.

**Results:**

All clinical outcome scores improved postoperatively in the older age groups for both procedures. Scores for the pain and pain-related subscale of the SAFE-Q improved by 37 points (*p* < 0.001) in post-total ankle arthroplasty patients and by 35 points in post-ankle arthrodesis patients (*p* < 0.001). Furthermore, multivariate analysis showed that the improvements observed in all scores were not different between the older and younger age groups for both post-total ankle arthroplasty and post-ankle arthrodesis patients, except for the SAFE-Q physical functioning subscale score for post-ankle arthrodesis patients. The clinical outcomes improved significantly in post-total ankle arthroplasty and post-ankle arthrodesis patients aged ≥ 75 years. Moreover, these improvements were similar to those observed in patients aged < 75 years.

**Conclusions:**

Surgical treatment of end-stage ankle osteoarthritis can be a viable treatment option, even in elderly patients, and can be expected to improve similarly to younger patients.

**Supplementary Information:**

The online version contains supplementary material available at 10.1186/s13018-023-03734-4.

## Background

End-stage ankle osteoarthritis (OA) is a disabling condition in which quality of life impairment is as severe as that associated with hip OA [1]. Although the risk of developing ankle OA is lower than that of developing OA in the hip and knee joints, the number of patients with ankle OA is expected to increase [2]. Surgical treatment for end-stage OA includes either total ankle arthroplasty (TAA) or ankle arthrodesis (AA) [3]. Both techniques have advantages and disadvantages, but their overall satisfactory clinical outcomes are similar [3]. Over the past decade, the performance of both surgical procedures has increased in Japan and worldwide [4, 5]

With increasing life expectancy worldwide, the global population is aging rapidly, especially in Japan, where the average life expectancy was > 80 years in 2019 [6]. The number of surgical treatments for degenerative joint diseases in elderly patients has been increasing accordingly; therefore, patients in their 70 s and 80 s commonly undergo joint arthroplasty [7]. However, limited data are available regarding whether clinical outcomes after TAA and AA in elderly patients are comparable to those in younger patients. Elderly patients may have coexisting medical conditions and reduced physical functioning, which hinders postoperative functional recovery. Several studies have compared the clinical outcomes of TAA between older and younger age groups [8–13]. However, these studies only included patients up to age 50–60 years, highlighting the outcomes in patients only of this age group and not in older patients. Few studies have reported the outcomes for older patients in their 70 s and 80 s [14, 15]. Additionally, since patients undergoing AA are generally young, only a few studies have assessed the clinical outcomes of AA in elderly patients [16, 17]. Data on the outcomes of TAA and AA may provide important information for clinical decision-making in our aging population.

Therefore, this study aimed to clarify the clinical outcomes of surgical treatment for ankle OA in elderly patients and to compare the results with those in younger patients. We hypothesized that the clinical outcomes of either the TAA or fusion technique will be the same in older and younger patients.

## Methods

### Study design

This multicenter, retrospective study was approved by the Ethics Committees of St. Luke’s International Hospital (17-R164) and seven other participating hospitals (four university hospitals and four district general hospitals) and was conducted in accordance with the Declaration of Helsinki. As this was a retrospective study, each hospital provided patients the option to opt out of the study, and no written consent was required. Patients were screened using data extracted from the electronic medical records of the eight participating hospitals. Patients who underwent TAA or AA for end-stage ankle OA between January 2015 and July 2019 were included in the study. The exclusion criteria were as follows: (1) diagnoses other than OA (e.g., rheumatoid arthritis and neuropathic arthritis), (2) revision surgery, (3) talocalcaneal joint arthrodesis, (4) history of TAA or AA on the contralateral side, and (5) < 1-year follow-up. Overall, 169 ankles of 169 patients met the inclusion criteria. Among these patients, three had rheumatoid arthritis, five underwent revision surgery, one had talocalcaneal joint arthrodesis, and one had a history of contralateral TAA or AA. Furthermore, 11 patients were lost to follow-up, including one who died. Therefore, the data of the remaining 148 patients, including 65 and 83 who underwent TAA and AA, respectively, were analyzed.

### Patient background

Patient background data, including age, sex, body mass index (BMI), and follow-up period, were recorded. Patients were classified into those aged ≥ 75 or < 75 years, with the threshold of 75 years used according to the definition set by the Ministry of Health, Labor, and Welfare of Japan to classify individuals as older senior citizens [18]. The radiographic severity of preoperative weight-bearing anteroposterior ankle radiographs was assessed using the Tanaka-Takakura classification [19]. We divided the patients into those with radiographic stage 3b or less (joint space obliteration extended to the roof of the talar dome) and those with stage 4 (obliteration of the whole joint space), as staging may affect clinical outcomes [20].

### Surgery

Surgery was performed by eight experienced foot and ankle surgeons (KA, SY, AT, TK, HS, TY, NK, and KN). The surgery type was classified as TAA or AA. Owing to the retrospective nature of this study, the operative indications and selection of surgical procedures were based on the patients’ and surgeons’ shared decisions. In general, TAA was performed in older, less obese, and sedentary individuals. In addition, patients with moderate deformities tended to undergo TAA. In contrast, AA was performed in younger, obese, and physically active patients as well as in those with severe deformities. Furthermore, the type of implant (TNK Ankle, Kyocera, Kyoto, Japan; Fine Ankle, Teijin Nakashima Medical, Okayama, Japan) was recorded in patients who underwent TAA. The surgical approach (arthroscopic or open) was recorded in patients who underwent AA.

### Clinical outcomes

Clinical outcomes were evaluated using the Japanese Society for Surgery of the Foot (JSSF) Hindfoot Scale and the self-administered foot evaluation questionnaire (SAFE-Q) [21, 22]. The JSSF hindfoot scale is a validated objective evaluation with subcales of 40 points for pain, 45 points for function, and 15 points for alignment, with higher scores indicating better clinical outcomes [22]. The SAFE-Q is a validated questionnaire comprising 34 questions on foot and ankle symptoms [21], and has five subscale scores: pain and pain-related, physical functioning and daily living, social functioning, shoe-related, and general health and well-being. The possible scores for each subscale range from 0 to 100, with higher scores indicating better clinical outcomes. We assessed the outcomes preoperatively and at the last follow-up, and the recorded improvement in scores was used for statistical analysis. Furthermore, we recorded the occurrence of revision surgery during the follow-up period. The major intraoperative complications investigated were fractures, technical errors, and iatrogenic complications including deep infections, peroneal tendon dislocation, and revision surgery.

### Statistical analysis

Patients who underwent TAA and AA were analyzed separately because the indications for these surgical procedures were different, as discussed earlier. Patient characteristics were compared between those aged ≥ 75 years and those aged < 75 years using Student’s t- and chi-square tests, as appropriate. Pre- and postoperative JSSF hindfoot scale and SAFE-Q subscale scores were compared using paired t-tests. To compare the clinical outcomes between the older and younger age groups, the recorded improvements in the JSSF and SAFE-Q subscale scores were also compared between the age groups using Student’s t-tests. The distribution-based minimal clinically important difference (MCID) was estimated by calculating 50% of the standard deviation of the change between the pre- and postoperative scores in this patient population. Complications were compared using Fisher’s exact test. Furthermore, to determine the independent association between age and clinical outcomes, analysis of covariance was performed. The clinical outcome was the objective variable, and age group was the explanatory variable, adjusting for sex, BMI, radiographic stage, and follow-up period. Statistical significance was set at *p* < 0.05.

### Patient and public involvement

This research was done without patient involvement. Patients were not invited to comment on the study design and were not consulted to develop patient-relevant outcomes or interpret the results. Patients were not invited to contribute to the writing or editing of this document for readability or accuracy.

## Results

### TAA

Sixty-five patients were analyzed, including 36 aged ≥ 75 years and 29 aged < 75 years (Table 1). Among these, 47 (65%) were women and only two (3%) were obese (BMI ≥ 30 kg/m^2^). The mean age of the older group was 78 ± 3 years. Except for age, the patient background did not differ significantly between the older and younger age groups (*p* = 0.18–0.82; Table 1). In the older age group, the JSSF hindfoot scale score improved from 53 points preoperatively to 90 points at the last follow-up (*p* < 0.001; Table 2). Similarly, all SAFE-Q subscale scores improved significantly, with improvements ranging from 30 to 37 points (*p* < 0.001 for all subscales; Table 2). The improvement in the scores for the five subscales was above the MCID estimates. The improvement of no outcome differed significantly between the older and younger age groups in the univariate analysis (Table 3). Furthermore, in the analysis of covariance adjusted for patient background, patient age was not associated with any clinical outcome (Table 3). In the older group, four patients (11%) had major intraoperative complications, including four fractures. In the younger group, two patients (6%) had major intraoperative complications, including two fractures. During the follow-up period, four patients (11%) in the older age group had major complications, including one deep infection that required debridement surgery, one peroneal tendon dislocation, and two revision surgeries due to an aseptic loosening and an infection. In the younger group, three patients (10%) had major complications, including one removal of impinging osseous overgrowth and two revision surgeries due to aseptic loosening. The occurrence of intra- and postoperative complications did not differ significantly between the groups (*p* = 0.57).

**Table 1:** Patient characteristics

	Total ankle arthroplasty			Ankle arthrodesis		
	< 75 (n = 29)	≥ 75 (n = 36)	*p*	< 75 (n = 61)	≥ 75 (n = 22)	*P*
Age (years)*	70 ± 4	78 ± 3	< 0.01	64 ± 9	79 ± 3	< 0.01
Sex (women / men)	20 / 9	27 / 9	0.61	31 / 30	15 / 7	0.21
Body mass index (kg/m^2^)*	23 ± 3	24 ± 3	0.18	25 ± 4	25 ± 3	0.71
Follow-up (months)*	26 ± 18	25 ± 15	0.82	18 ± 9	20 ± 9	0.23
Radiographic stage (≤ 3b / 4) **	17 / 12	21 / 15	0.79	22 / 39	10 / 12	0.31
Type of surgery (TNK Ankle / Fine Ankle) (Open / arthroscopic)	26 / 3	34 / 2	0.66	5 / 56	1 / 21	0.96

Values indicate the number of patients, unless indicated otherwise

*Mean ± standard deviation

**Tanaka-Takakura classification

**Table 2:** Pre- and postoperative clinical outcomes for total ankle arthroplasty according to patient age

	< 75 (n = 29)				≥ 75 (n = 36)			
	Pre	Post	MCID	*p*	Pre	Post	MCID	*p*
JSSF scale	53 ± 11	85 ± 14	8.7	< 0.01	53 ± 12	90 ± 12	8.3	< 0.01
SAFE-Q								
Pain and pain-related	46 ± 17	68 ± 24	12.8	< 0.01	40 ± 20	77 ± 19	10.1	< 0.01
Physical functioning	46 ± 18	68 ± 26	11.7	< 0.01	38 ± 23	68 ± 17	11.5	< 0.01
Social functioning	43 ± 25	70 ± 27	15.3	< 0.01	34 ± 32	69 ± 29	15.2	< 0.01
Shoe-related	58 ± 26	71 ± 31	14.2	0.04	45 ± 29	78 ± 19	11.2	< 0.01
General health	48 ± 24	72 ± 28	14.7	< 0.01	36 ± 27	75 ± 25	15.4	< 0.01

Values are presented as mean ± standard deviation

*JSSF* Japanese Society for Surgery of the Foot, *SAFE-Q* self-administered foot evaluation questionnaire, *MCID* minimal clinically important difference

**Table 3:** Comparison of clinical outcomes between patients aged < 75 and ≥ 75 years

	Total ankle arthroplasty				Ankle arthrodesis			
	< 75 (n = 29)	≥ 75 (n = 36)	p*	p**	< 75 (n = 61)	≥ 75 (n = 22)	p*	p**
JSSF scale	36 ± 17	37 ± 15	0.14	0.13	35 ± 17	32 ± 17	0.35	0.38
SAFE-Q								
Pain and pain-related	33 ± 19	37 ± 26	0.12	0.12	39 ± 26	34 ± 20	0.69	0.84
Physical functioning	26 ± 24	30 ± 25	0.79	0.80	29 ± 24	20 ± 23	0.02	0.04
Social functioning	28 ± 40	33 ± 41	0.90	0.94	40 ± 31	33 ± 31	0.09	0.12
Shoe-related	23 ± 33	31 ± 30	0.12	0.13	30 ± 29	26 ± 23	0.62	0.70
General health	31 ± 30	37 ± 30	0.56	0.63	39 ± 30	30 ± 32	0.08	0.11

Values represent the mean ± standard deviation of improvement in score

*JSSF* Japanese Society for Surgery of the Foot, *SAFE-Q* self-administered foot evaluation questionnaire

*Student’s t-test

**Analysis of covariance adjusted for patient characteristics

### AA

Eighty-three patients were analyzed, including 22 aged ≥ 75 years and 61 aged < 75 years. Except for age, the patient background did not differ significantly between the older and younger age groups (*p* = 0.21–0.96, Table 1). The proportion of women was 55% (n = 46), and the mean BMI was 25 kg/m^2^, with five (6%) patients recorded as obese. The mean age was 79 ± 3 years in the older age group and 64 ± 9 years in the younger age group. In the older age group, the JSSF hindfoot scale and SAFE-Q subscale scores improved postoperatively (Table 4). Specifically, SAFE-Q scores improved by 20–35 points (*p* < 0.001 for all subscales; Table 4). The improvement in the scores for the five subscales was above the MCID estimates. Similar to the patients who underwent TAA, the improvement of all clinical outcomes did not differ significantlybetween the older and younger age groups after univariate analysis (Table 3). In the analysis of covariance adjusted for patient background, patient age was not associated with any of the clinical outcomes, except for the SAFE-Q physical functioning subscale score (*p* = 0.04, Table 3). The recorded improvements in physical functioning scores were 20 and 29 points in the older and younger age groups, respectively. In the older group, none of the patients experienced major intraoperative complications. In the younger group, two patients (6%) had major intraoperative complications, including iatrogenic complications (endosseous drill breakage). During the follow-up period, one (4%) patient in the older age group had the major complication of revision surgery due to non-union. In the younger age group, three (5%) patients had major complications, including one revision surgery due to non-union overgrowth and two revision surgeries due to aseptic loosening. The occurrence of intra- and postoperative complications did not differ significantly between the groups (*p* = 0.62). The ratios of TAA and AA surgeries at each of the eight hospitals were determined (Additional file 1: Table S1). Moreover, comparisons of the clinical results before and after TAA or AA surgery in hospitals grouped by large and small number of cases, showed that both groups improved significantly in most scales (TAA: Additional file 2: Table S2 and Additional file 3: Table S3; AA: Additional file 4: Table S4 and Additional file 5: Table S5).

**Table 4:** Pre- and postoperative clinical outcomes for ankle arthrodesis depending on patient age group

	< 75 (n = 61)				≥ 75 (n = 22)			
	Pre	Post	MCID	*p*	Pre	Post	MCID	*p*
JSSF scale	53 ± 16	89 ± 10	8.7	< 0.001	53 ± 12	90 ± 12	8.3	< 0.001
SAFE-Q								
Pain and pain-related	43 ± 17	82 ± 22	12.8	< 0.001	45 ± 18	80 ± 15	10.1	< 0.001
Physical functioning	47 ± 20	76 ± 22	11.7	< 0.001	43 ± 27	63 ± 22	11.5	< 0.001
Social functioning	40 ± 28	81 ± 27	15.3	< 0.001	36 ± 27	69 ± 33	15.2	< 0.001
Shoe-related	48 ± 27	77 ± 24	14.2	0.04	47 ± 25	74 ± 26	11.2	< 0.001
General health	40 ± 26	79 ± 27	14.7	< 0.001	37 ± 26	67 ± 32	15.4	< 0.001

Values are presented as mean ± standard deviation

*JSSF* Japanese Society for Surgery of the Foot, *SAFE-Q* self-administered foot evaluation questionnaire, *MCID* minimal clinically important difference

## Discussion

The present study showed that surgical treatment for end-stage ankle OA resulted in satisfactory clinical outcomes in patients aged ≥ 75 years, and improvements in the scores were observed in patients undergoing both TAA and AA. Furthermore, these improvements were comparable with those observed in patients aged < 75 years. Our results suggest that surgical treatment of end-stage ankle OA is a viable treatment option, even in elderly patients.

Moreover, the JSSF hindfoot scale score and all SAFE-Q subscale scores improved significantly for patients with TAA in the older age group, with a mean age of 78 years. Specifically, the improvement in SAFE-Q subscale scores ranged from 13 to 38 points. Nonsurgical treatment is the first-line treatment for ankle OA; however, insufficient data is available to support its efficacy [23]. Moreover, elderly patients aged > 75 years tend to undergo nonsurgical management because of their limited physical functioning and concern for postoperative complications [7]. The MCID in the SAFE-Q scores of the patients who underwent TAA were not determined. However, improvements identified in this study were clinically significant and comparable with those described in previous reports [24–26].

This study further showed that the improved outcomes in the older age group were comparable with those in the younger age group of patients who underwent TAA. Our results conform with those of Tenenbaum et al. [14], in which patients aged > 70 years and those aged 50–60 years demonstrated similar improvement in both the American Orthopedic Foot and Ankle Society (AOFAS) ankle/hindfoot scale and visual analog scale pain scores. Demetracopoulos et al. [15] also reported that most of the clinical outcomes in patients aged > 70 years were comparable with those in patients aged ≤ 70 years, although the AOFAS function and SF-36 vitality subscale scores were lower in older than in younger patients. Other studies used a younger cutoff age of 50–65 years [8–13], and reported no difference in clinical outcomes according to age. The results of our study suggest that TAA could be the treatment of choice, even in patients aged ≥ 75 years. Furthermore, the intraoperative and postoperative complications did not differ between the two groups, which is a major concern in the elderly population.

Similar to TAA, all clinical outcomes improved postoperatively in patients who underwent AA in the older age group (mean age, 79 years). Few studies have reported the postoperative results for AA in geriatric patients [16, 17]. Strasser et al. [17] found that the postoperative foot and ankle ability measurement score was 82 points in 22 patients with a mean age of 75 years. However, those authors [17] did not obtain preoperative scores. Additionally, Yang et al. [16] assessed 41 patients (mean age, 71 years) with arthroscopic AA and observed significant improvements in the AOFAS ankle/hindfoot scale and visual analog scale pain scores. Although a direct comparison would be difficult because of the different evaluation measures used, our results were consistent with those of previous studies [16, 17].

After AA, improvements in clinical scores were comparable between patients aged < 75 and ≥ 75 years. The age at surgery is reportedly not associated with clinical outcomes [27–29]. However, the patients in previous studies were relatively young with a mean age of 57–63 years. The results of our study suggest that even older patients can expect similar levels of clinical improvement after AA. In contrast, Berlet et al. [30] reported that patients aged ≥ 60 years had a higher risk of non-union after foot and AA. Nevertheless, we could not determine the effect of age on bone union due to the small sample size. Furthermore, there were no differences in intraoperative and postoperative complications between the groups or in the TAA group.

This study had several limitations. First, the older age group might have consisted of selected patients with good health status; therefore, surgeons would have logically expected good postoperative outcomes. Although age is considered a risk factor in ankle joint surgery [31], the preoperative assessment of an individual's condition should be based on various aspects, together with age. Previous reports suggest that preoperative performance status is more important than age in hip arthroplasty [32]. Thus, the risk assessment of performance status on postoperative clinical outcomes requires further investigation. This limitation could have resulted in a bias toward better results in the older age group. Second, several covariates that may affect clinical outcomes, including comorbidities, mental status, and physical activity level, were not included in the multivariate analysis because of the retrospective nature of the study. Although our study provides clinically meaningful information for patients and surgeons, further prospective studies are necessary to draw definitive conclusions regarding the association between age and clinical outcomes. Third, the follow-up period was relatively short. However, it is important to consider both short- and long-term outcomes in elderly patients, regardless of their shorter life expectancy than younger patients. Fourth, despite collecting patient data from eight hospitals, the study population was relatively small, especially when comparing the incidence of revision surgery between age groups.

## Conclusions

The JSSF scale and all SAFE-Q subscale scores improved postoperatively in patients with end-stage ankle OA aged ≥ 75 years. Furthermore, the recorded improvements in the scores were comparable with those in patients aged < 75 years. Surgical treatment of end-stage ankle OA using either TAA or AA is a viable treatment option, even in elderly patients. Therefore, elderly patients should not be excluded from surgical management owing to their age.

## Supplementary Information

**Additional file 1**. **Table S1**: The ratio of total ankle arthroplasty and ankle arthrodesis performed by eight operators at eight facilities.

**Additional file 2. Table S2:** Comparison of the JSSF and SAFEQ in the TAA<75 and TAA≧75 Groups of hospitals with a large number of cases.

**Additional file 3. Table S3:** Comparison of the JSSF and SAFEQ in the TAA<75 and TAA≧75 Groups of hospitals with a small number of cases.

**Additional file 4. Table S4:** Comparison of the JSSF and SAFEQ in the AA<75 and AA≧75 Groups of hospitals with a large number of cases.

**Additional file 5. Table S5:** Comparison of the JSSF and SAFEQ in the AA<75 and AA≧75 Groups of hospitals with a small number of cases.

## Data Availability

Not applicable.

## Abbreviations

OA: Osteoarthritis
TAA: Total ankle arthroplasty
AA: Ankle arthrodesis
BMI: Body mass index
JSSF: Japanese Society for Surgery of the Foot
SAFE-Q: Self-administered foot evaluation questionnaire
MCID: Minimal clinically important difference
